# The Practical Significance of Perinatal Cardiology Based on Own Experience

**DOI:** 10.34763/devperiodmed.20182203.229237

**Published:** 2018-10-04

**Authors:** Maria Respondek-Liberska

**Affiliations:** 1 Department of Diagnostics and Prevention of Congenital Defects, Medical University of Lodz, Lodz Poland; 2 Department of Prenatal Cardiology Polish Mother’s Memorial Hospital, Research Institute (ICZMP) in Lodz, Lodz Poland

**Keywords:** fetal heart defect, cardiac surgery of the newborn, classification of fetal heart defects, fetal treatment, fetal surgery, wada serca płodu, operacja kardiochirurgiczna u noworodka, podział wad serca płodu, leczenie płodu, zabiegi u płodu

## Abstract

The article underlined the role of perinatal cardiology in contributing to the medical care of the pregnant woman taking into consideration the technical and medical progress which has made it possible to save not only human life but also its quality. The role of correct early diagnosis, as well as correct treatment was discussed and the results of such procedure demonstrated.

## Streszczenie

Biorąc pod uwagę ogromny postęp techniczny i nauk medycznych pozwalający nie tylko na ratowanie życia, ale także jego jakości, przedstawiono w pracy rolę perinatalnej kardiologii w ramach opieki medycznej nad kobietą ciężarną. Omówiono postępowanie w okresie pre- i perinatalnym z punktu widzenia wczesnego rozpoznania i wczesnego leczenia. Omówiono również wyniki tego postępowania.

## Introduction

The challenge of today’s medicine is not only to save human life but also its quality. This plays a special role in children. Cardiac malformations are the first cause of mortality in term newborns. This problem requires more attention and improvement in this field is necessary. Therefore, the basic issues are as follows: early and correct prenatal diagnosis, early and correct treatment (including in utero), the effective, complex multidisciplinary treatment of newborn’s and infants. There is a great need for all of these [1, 2, 3, 4, 5, 6, 7, 8, 9, 10, 11].

The aim of this paper is to illustrate the role of perinatal cardiology in the procedure of early, perinatal diagnosis and the early perinatal treatment of fetuses, newborns and children on the basis of the author’s experience.

## Material, methods, results

For this review, the data from completed case histories of fetuses’ and newborn’s and infants diagnoses with fetal cardiological malformations were obtained from the files of the Department of Prenatal Cardiology, at the Institute Polish Mother’s Memorial Hospital, Research Institute (ICZMP), Lodz. Ten cases were selected to illustrate the major problems.

### Case 1

In the first and second trimester of pregnancy no abnormalities were detected in a 36-year-old healthy pregnant women under prenatal medical care. In the third trimester (the 32nd week of gestation), a heart malformation (single ventricle) was detected in our Department. According to the classification of cardiac malformations in prenatal cardiology, it was classified as a ”severe planned cardiac malformation” (Figure 1). Delivery of the newborn with heart defect and ductal dependent circulation was scheduled in our center the ductal dependent was planned. Postnatal echocardiography confirmed the prenatal diagnosis and prostin infusion was started just after birth. The newborn underwent cardiac surgery in the form of the Norwood procedure with the excision of the interventricular septum on the 6^th^ day of postnatal life. The newborn was discharged in the 27th day of postnatal life in a generally good condition. The consecutive steps of further procedures were planned for the 6th month of life, in the form of the bidirectional Glenn procedure and the Fonatan procedure in the 2nd – 3rd year of life. Currently, the clinical status of the 7-year-old child is very good. (Figure 1a).

**Fig. 1:**
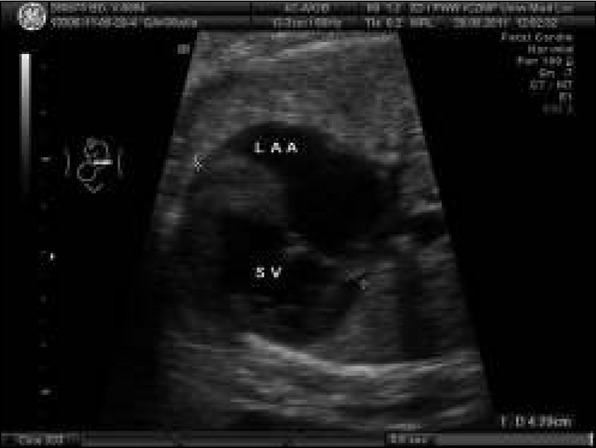
Abnormal fetal heart (fetus in cephalic presentation, longitudinal position I): common atrium with left atrial appendage (LAA) on the right side of the fetal heart, single atrioventricular valve opening to single ventricle with trabeculation suggesting right ventricle. Ryc. 1. Nieprawidłowy obraz serca płodu (płód w położeniu główkowym, postawa I): szeroki dominujący przedsionek z uszkiem sugerującym uszko lewego przedsionka LAA, które leży po prawej stronie serca pojedyncza komora o typie komory prawej (RV), jedna zastawka przedsionkowo-komorowa.

**Fig. 1a:**
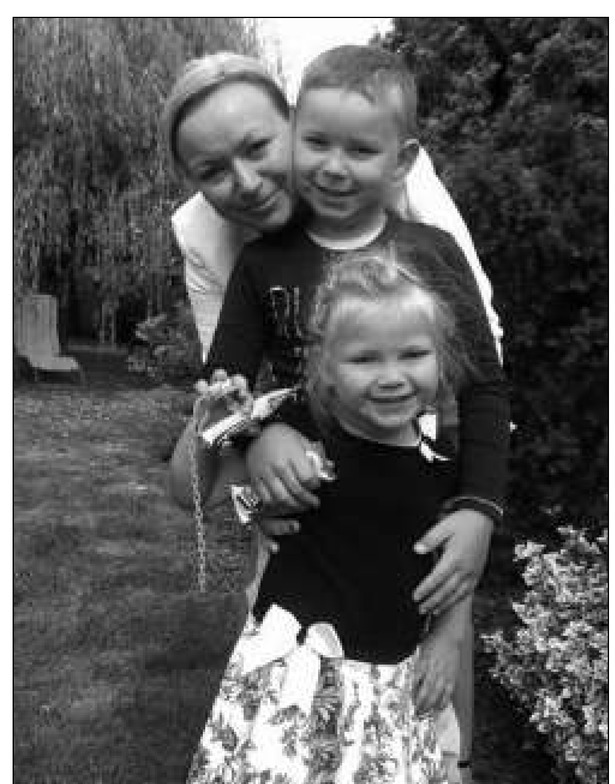
Contemporary photo of our patient, courtesy of her parents and the family. Ryc. 1a. Współczesne foto dziewczynki z rodziną (dzięki uprzejmości rodziców).

### Case 2. Total anomalous pulmonary venous connection (TAPVC)

**Fig. 2:**
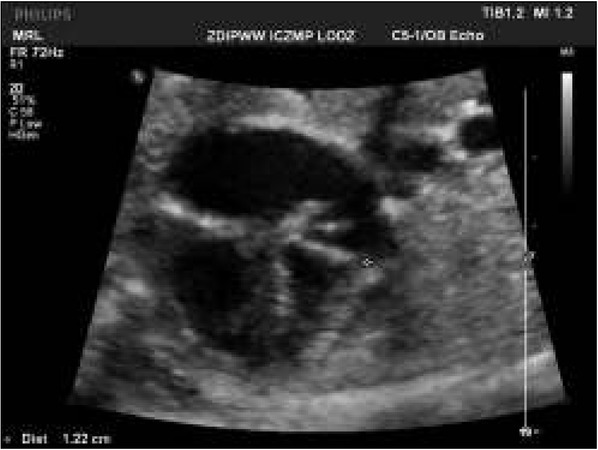
Abnormal 4 chamber view of the fetal heart. Disproportion at the level of the atria and ventricles. Ryc. 2. Nieprawidłowy obraz 4 jam serca. Dysproporcja na poziomie przedsionków i komór.

This was the first pregnancy of a healthy woman. She was under obstetric care and during the first and second trimester no abnormalities were detected. In the 38th week, abnormalities of the fetal heart in the four-chamber view were detected in the form of atrial and ventricular disproportion (Figure 2). The coarctation of the aorta or hypoplasia of the transverse aortic arch was suspected by the obstetrician. The mother was referred to our center for a consultation. The following abnormalities were detected: the right atrium and the right ventricle were enlarged in comparison to the left atrium and the left ventricle. In contrast, the heart diameter, the thickness of its wall and contractility were within normal limits. The aortic arch was continuous. The cardiac capacity measured on the CVPS scale was 10 points. Echocardiographic examination included systemic and pulmonary venous drainage; the systemic veins drained to the right atrium, while pulmonary veins showed an atypical spectrum of flow and the absence of connection of pulmonary veins with the posterior wall of the left atrium. An atypical venous vessel located posteriorly to the posterior cardiac wall was detected and this vessel drained to the ductus venosus (Aranti). The malformation was classified as **critical** and in urgent need for cardiac surgery.

The package of prenatal findings was provided to the team of the cardiac surgeon, a neonatologist, an obstetrician and a pediatric cardiologist. The mother delivered a newborn weighting 3100 g whose prenatal diagnosis was confirmed. The following day, after changing the earlier planned surgeries, the newborn underwent a total correction of the malformation. Currently, the boy is 2 years old, developing normally and does not need any pharmacological support.

### Case 3. Cardiac ectopy

**Fig. 3:**
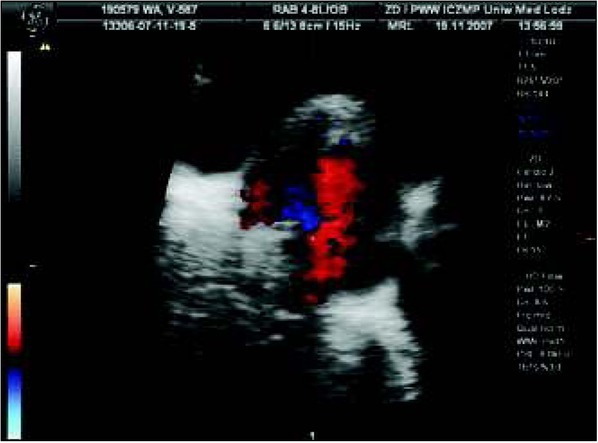
Fetal heart outside the fetal chest. Ryc. 3. Obraz serca płodu poza klatką piersiową.

The first pregnancy in a young healthy woman. At 13 weeks pregnant the obstetrician detected cardiac ectopy and suggested amniocenthesis and termination of the pregnancy, which is a legal procedure in Poland (Figure 3). The patient decided to continue the pregnancy and asked our Department for a consultation. We confirmed the early diagnosis from the first trimester and found no other abnormalities. In the 24th week of pregnancy we observed an alteration of the heart’s position with the atria „digging” into the chest wall, at the level of the fetal sternum. The fetal heart had two symmetrical atria, two symmetrical ventricles and two big vessels, equal in size. Echocardiographic monitoring had been performed on outpatient basis until the 37th week of pregnancy, then the pregnant woman was admitted to our hospital. Immediately after cesarean section the newborn was transported to the cardiac surgery unit. The heart was reinstated in the chest. A band was posited on the enlarged pulmonary artery. The newborn was discharged from the intensive care unit on the 4th day following cardiac surgery, and discharged home at the age of 5 weeks of postnatal life.

At the age of 10 months the sudden death of the infant occurred.

Two years later his mother was pregnant again, fetal heart echocardiography was performed 3 times revealing no structural or functional abnormalities and a healthy newborn was born at term.

### Case 4. Critical aortal stenosis

**Fig. 4:**
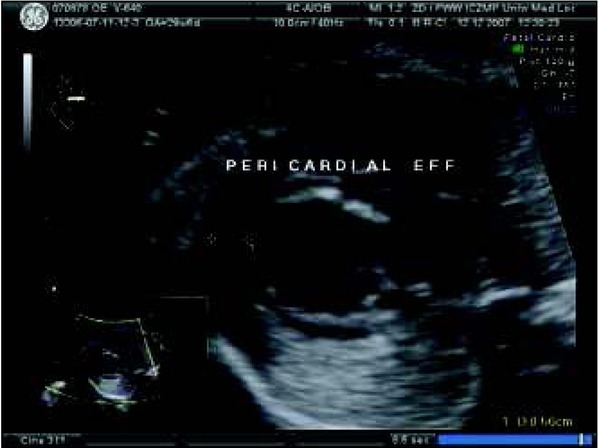
Abnormal view of the fetal heart at 29th week of gestation with pericardial effusion of 5 mm – one of the signs of congestive heart failure and fibroelastosis of enlarged left ventricle. Ryc. 4. W 29 tyg. nieprawidłowy obraz serca płodu z cechami niewydolności krążenia – wysięk w osierdziu 5 mm oraz fibroelastoza powiększonej lewej komory serca.

There were no sonographic abnormalities detected in the first trimester of the pregnancy. Polyhydramnion, accompanied by cardiac enlargement and an abnormal four chamber view with cardiac effusion and fibroelastosis of left ventricle were observed in the 29th week (Figure 4). Critical aortic stenosis with eminent congestive heart failure was diagnosed on the basis of fetal echocardiography. The left ventricle was akinetic with features of fibroelastosis, without blood inflow. The flow through the foramen ovale was redirected left to right. Prenatal treatment with maternal digitalis was initiated: first intravenously, then orally. After 8 weeks, fetal development and fetal cardiac size normalized. At the age of the 38th week of gestation, the heart area/chest area ratio (HA/CA) decreased from 0.6 up to 0.45 but the left ventricle remained akinetic.

Cesarean delivery was planned for the 38th week for the critical dependent ductal heart defect with subsequent balloon valvuloplasty. THE LATTER WAS PERFORMED 2 HOURS AFTER DELIVERY.

This procedure was performed again on the 21th day of life due to the increasing of blood flow gradient through the aortic valve. The boy was discharged home with a gradient 25 mm HG, a trace of aortic valve insuffciency and left ventricular shortening fraction of 30%. Currently at the age of 8 years his aortal valve gradient is 20 mm HG and the child remains without need for pharmacotherapy.

### Case 5. Supraventricular tachycardia (SVT)

**Fig. 5:**
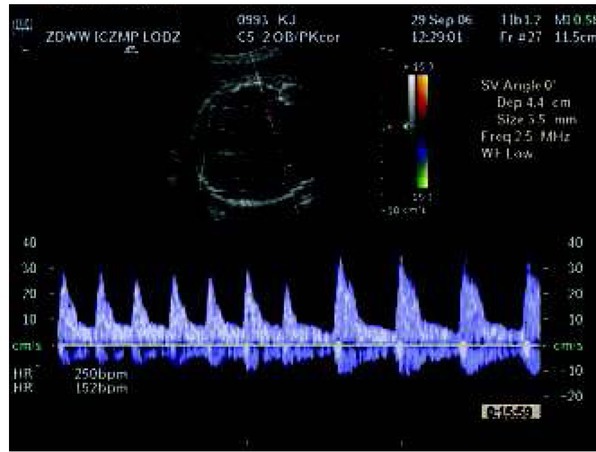
Abnormal fetal heart rhythm with supraventricular tachycardia. Ryc. 5. Nieprawidłowy rytm serca z wstawką częstoskurczu.

Following unremarkable results in the 1st and 2nd trimester of pregnancy, an obstetrician detected ascites in the peritoneal cavity of the fetus and referred the patient to the Obstetric Clinic at the IPMHC. Exudate in the peritoneal cavity was followed by exudate in the pleural cavity. The fetus was referred for echocardiography, before planned evacuation of the effusion. Echocardiography confirmed normal heart structure but registered fetal tachycardia of 240 beats /min with atrioventricular conduction 1:1 (Figure 5): 1. Taking into account that disturbances in the atrioventricular conduction are the cause of effusion in the body cavities, we suggested transplacental administration of digoxin, first intravenously then orally, which lasted 10 weeks. After 2 weeks of therapy, no effusions were observed and after 3 weeks, no tachycardia was recorded.

The boy was born by spontaneous full-term labor without any abnormalities and after 7 days in the hospital he was discharged. The boy is now 5 years old and is developing normally.

### Case 6. Complete heart block

**Fig. 6:**
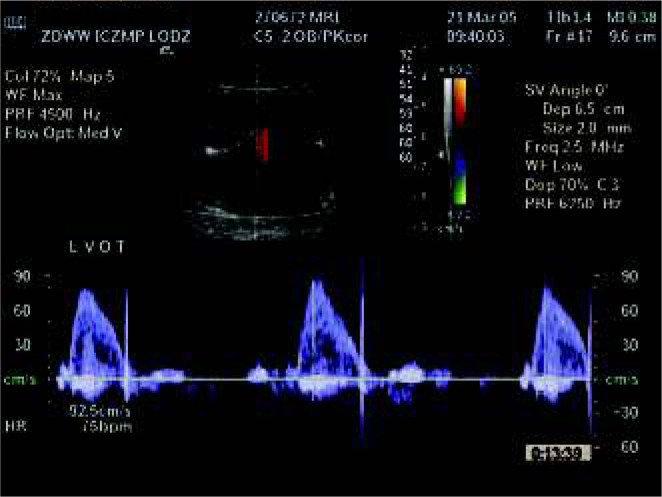
Isolated complete heart block in Doppler evaluation in a fetus with normal heart anatomy. Ryc. 6. Izolowany blok serca płodu (przy prawidłowej budowie serca) w badaniu za pomocą Dopplera.

It was the first pregnancy of a young woman. In the 20^th^ week of gestation, enlargement of the fetal heart accompanied by slow heart rate was observed; the pregnant woman was referred to our unit. Using ultrasonography, we detected a normal placenta and a normal volume of amniotic fluid. Normal biometry and normal cardiac anatomy, accompanied by abnormal heart rate – atrial, 140 beats /min, while ventricular rate was 80 beats / min (Figure 6). Atrioventricular block was diagnosed but because intracardiac, peripheral (umbilical artery and veins and cerebral media) blood flows were normal and there were no signs of circulatory insufficiency, no treatment was instituted. As atrioventricular block may be the first sign of collagenosis in the pregnant woman, the test for anti Ro SS-A and ani Ro SS- B (Sjogren A syndrome) was suggested. Anti-Ro abs were detected but no clinical symptoms of collagenosis. The full diagnosis of the pregnant woman was established in the 24^th^ week of pregnancy and corticosteroids were initiated orally.

A full-term girl was born weighing 3200 g, APGAR 9. A complete block with the ventricular rate of 80/ min was confirmed by ECG and initially orciprenaline was started but no effect was observed. The child was discharged indicating the possibility of the implantation of a pacemaker in the future. At the age of 3 years, no signs or symptoms of circulatory insuffciency is observed.

### Case 7. Congenital heart defect with complete heart block

**Fig. 7:**
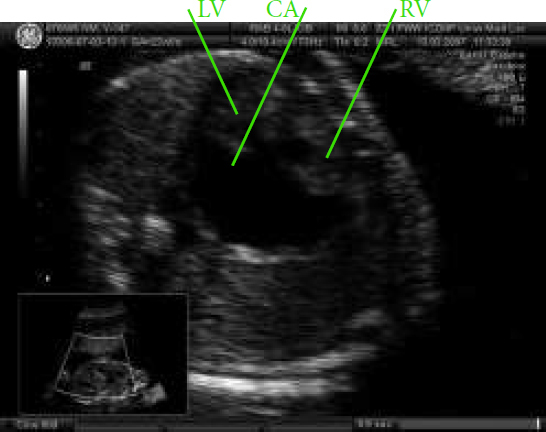
Fetal heart defect and complete heart block in a case of prenatal left isomerism, CA – common atrium, RV – right ventricle, LV – left ventricle. Ryc. 7. Obraz wady serca powikłanej blokiem całkowitym w przebiegu izomeryzmu lewostronego: CA wspólny przedsionek, RV – prawa komora, LV – lewa komora, PA.

It was the second pregnancy in a young woman. Two years earlier her healthy boy was born. Until the 16^th^ week, ultrasonography revealed no abnormalities. At 16 weeks of pregnancy, an abnormal cardiac four chamber view was detected and amniocentesis and genetic investigation was performed; the 46XY was found. Based on echocardiography in our unit, cardiomegaly and a complex malformation of the common atrioventricular canal with a common atrium, left isomerism and complete cardiac block were diagnosed (Figure 7). Such a syndrome is classified as one of the most severe cardiac anomalies, for which currently no prenatal treatment is available. The pregnant woman took the decision to deliver in a local hospital. In another academic center, a cesarean section was performed and the newborn was trans-shipped to our hospital to the Department of Newborn’s Intensive Care. Postnatal cardiology confirmed the prenatal diagnosis; conservative care was suggested. The child died on the 21st day of postnatal life.

### Case 8. Congenital heart defect and extracardiac malformation

**Fig. 8:**
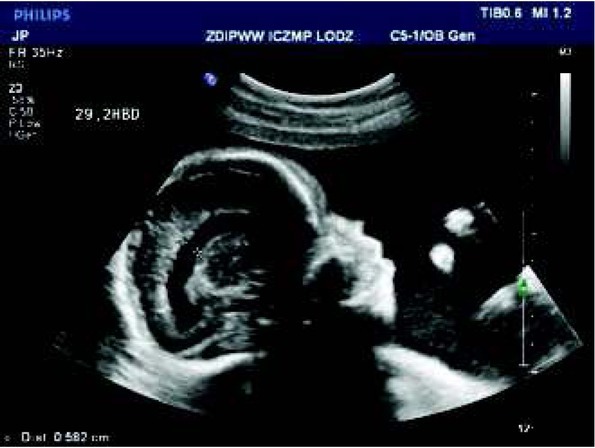
Abnormal fetal profile: short nasal bone, rentriculo-megaly in addition to heart defect suggested poor autcome Ryc. 8. Nieprawidłowy profil twarzy płodu, krótka kość nosowa, poszerzenie komór bocznych u płodu z wadą serca sugerują poważne rokowanie dla noworodla.

Fetal nuchal translucency was detected in a pregnant woman aged 31 years. The investigation after amniocentesis revealed 46XY. At the age of 18 weeks of pregnancy, a severe cardiac malformation of pulmonary valve atresia was detected and the pregnant woman was sent for cardiac surgery counselling. A cardiac surgeon suggested the outline of the surgical procedure. In the 23rd week of pregnancy, echocardiography was performed in our unit, and a diagnosis of pulmonary valve atresia with partial ventricular septal defect was diagnosed. Additionally, polyhydramnios, enlargement of cerebral ventricles dysmorphic features (Figure 8), hypoplasia of the vermis, hypoplasia of the thymus were detected. The pregnant woman was informed of the possibility of premature delivery and poor prognosis. In the 29^th^ week of pregnancy, cardiomegaly, pleural and peritoneal effusion and change in the cardiac axis was seen; these abnormalities stressed that the fetus’ condition in the course of pregnancy may change in time. In our case, the planning of cardiac surgery in the middle of gestation was unreliable. The newborn died just after birth.

### Case 9. A stent in the right fetal atrium

**Fig. 9:**
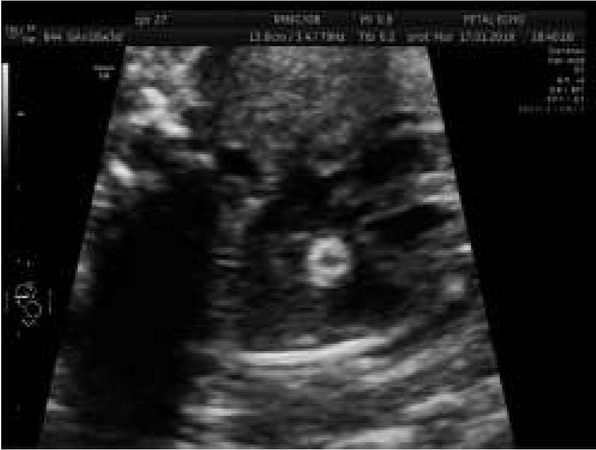
Chamber view of the fetal heart with the foreig body in right atrium (hyperechogenic ring „white”). Ryc. 9. Obraz 4 jam serca płodu z ciałem obcym w świetle prawego przedsionka „biały”.

A fetus was sent to our unit in Lodz after an attempt of prenatal procedure in the 22nd week of pregnancy. In the first trimester, nuchal translucency up to 3.5 mm was detected. Amniocentesis and cytogenetic investigation was performed and the result was 46XY. In the 18^th^ week of pregnancy, a cardiac malformation was detected – closed interatrial septum, ventricular asymmetry with the right ventricle larger than the left, numerous muscular interventricular septal defects, persistent vena cava superior and pulmonary flow abnormalities with evident mitral valve regurgitation. An attempt to insert a stent into the interatrial septum performed in another hospital was unsuccessful and the stent remained within the right atrium (Figure 9).

In our unit at the IPMHC, at 28^th^ week of pregnanty, we visualized a heart of normal size with two asymmetrical ventricles; the right ventricle larger than the left one and a hypoplastic but complete aortic arch. Intracardiac flows were normal, including the tricuspid valve despite the presence of a stent in the right atrium. The persistent superior vena cava resulted in the narrowing of the aorta arch but the flow in the aorta remained normal. At 32^nd^ weeks of pregnancy , spontaneous rupture of membrane was observed and delivery by cesarean section was performed in Warsaw hospital. The premature newborn weighing 1300 g was transferred to the Children’s Memorial Health Institute, Warsaw; after an attempt of cardiac surgery on day 6 of postnatal life, newborn died a few hours later.

### Case 10. Extracardiac malformation with a normal heart

**Fig. 10:**
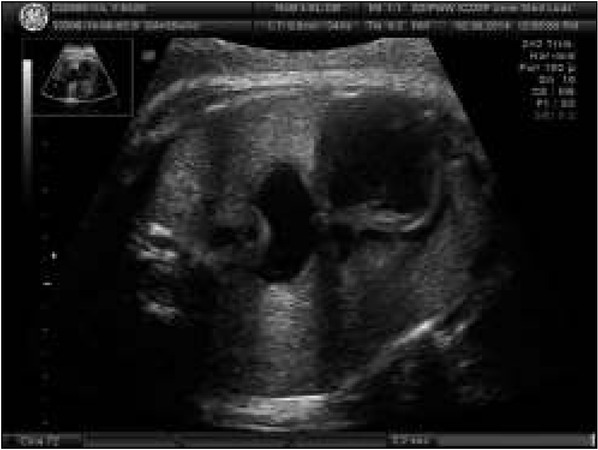
Diaphragmatic hernia and normal heart anatomy. Ryc. 10. Przepuklina przeponowa płodu i prawidłowa budowa serca.

Practically all extracardiac malformations require prenatal echocardiography to establish full diagnosis and prognosis. For prenatal therapy only, cases of normal cardiac structure and function are qualified, while those with multiple malformations – i.e. combinations of cardiac and extracardiac malformations are characterized by poor prognosis (Figure 10). On the other hand, those cases of extracardiac malformations and normal heart are characterized by much better prognosis, provided that pregnancy is ended at term.

## Discussion

In case 1, the wrong diagnosis (missed heart defect) was made in the first and second trimester – two times the malformation was missed. The correct prenatal diagnosis of the single ventricle and cardiac malformation changed the planned place of delivery. After the proper diagnosis, a safer and cheaper means of fetal transportation – via in utero was used to avoid transshipment of the newborn. The newborn was enrolled for planned cardiac surgery before clinical deterioration.

Before the time of prenatal cardiology, such a malformation was classified as lethal and newborns usually died before arrival at the cardiac surgery unit [6]. Currently newborns with prenatal diagnosis rarely die, because there is enough time to perform the cardiac catheterisation procedure or planned cardiac surgery as early as possible.

Case 2 illustrates that the alertness of the obstetrician enabled the suspicion of a cardiac malformation and the pregnant woman was sent for specialized echocardiographic evaluation. Echocardiography performed at 38 weeks of pregnancy made it possible to diagnose a rare malformation, not found in the first or the second trimester. The diagnosis led to delivery at the IPMHC and to accelerating cardiac surgery in the still asymptomatic newborn [12].

A similar case of a cardiac malformation but without either prenatal or perinatal diagnosis was admitted to our hospital in the 4^th^ month of life in a critical condition and with renal insuffciency. The child was saved despite the late diagnosis but his stay at the intensive care unit lasted 3 months. Prenatal diagnosis of anomalous pulmonary venous connection accompanied by venous stenosis is currently regarded as a lethal malformation [13], while partial anomalous venous connection is not life-threatening [14].

In case 3 with ectopia cordis, prenatal echocardiographic monitoring enabled full term delivery and fast transport from the obstetric unit to the cardiac surgery unit of the same hospital. The ectopia cordis was regarded a lethal malformation [15, 16]. A description of this case was published separately in 2011 [17], while later on the survival of a newborn with the Cantrell syndrome (ectopia cordis accompanied by omphalocele) from our hospital was also reported [18].

In case 4, prenatal detection of a cardiac malformation in the course of pregnancy made it possible to send the pregnant woman to a reference center, where, after the diagnosis of critical aortic stenosis, and an attempt of transplacental therapy and echocardiographic monitoring revealed the normal development of the fetus. As a result, cardiac surgery was avoided and the reversing of fibroelastosis and regeneration of the left ventricle was achieved following newborn’s balloon valvuloplasty.

In our hospital balloon valvuloplasty in a fetus was attempted in the past but usually ended with premature delivery; we stopped these procedures and performed balloon valvuloplasty in newborns as soon as possible. Such a procedure was successful also in other cases [19, 20, 21, 22, 23].

Case 5; Fetal supraventricular tachycardia of >220/ min may be diffcult to spot because of the paroxysmal nature unnoticeable for both the pregnant woman and the obstetrician. Initiation of transplacental therapy made it possible to control arrhythmia and exudates. The pregnancy was safely continued and the child developed normally. These cases illustrate the point that every exudate is a reason to perform fetal echocardiography. It is worth mentioning that fetal therapy for arrhythmias has been used for 30 years [24, 25, 26, 27].

In case 6 a total cardiac block was diagnosed at a relatively late prenatal period. The cause of the damage to the atrioventricular node was the presence of maternal antibodies which cross the placenta in the 15^th^-16^th^ week of pregnancy. Perhaps if bradycardia or the first degree of atrioventricular block (time of atrioventricular (PR interval >140 msec) had been detected earlier and corticosteroid therapy had been initiated before the 20-24th week of gestation, the total arterioventricular block would not have developed [28].

In case 7, the obstetrician detected a complex cardiac malformation of the common atrioventricular canal and suspected Down’s syndrome, so he suggested cytogenetic evaluation. If he had been aware that in left isomerism the results of cytogenetics are always normal, perhaps we would have skipped the amniocentesis. In this case, exposing the fetus to a miscarriage was not justified. With such a prenatal diagnosis, the transport of the newborn to an intensive care unit (300 km) was also unjustified. A newborn with a severe cardiac malformation, even with a total heart block, is born in a good general condition. The latter probably misled the neonatologists who organized shipment to the reference hospital. It is worth remembering that medical proceedings should be different in the case of an isolated cardiac block and a block accompanied by heart malformation [29, 30, 31].

Case 8. Prenatal cardiology is not merely important for the correct diagnosis of cardiac malformation but also for correct monitoring and prediction of the neonatal prognosis. The manifold course of the second half of pregnancy taught prenatal cardiologists that early planning of the mode of delivery or prediction of the clinical status of a newborn based on findings in midgestation is unreliable. The advice given to a pregnant woman in the middle of pregnancy cannot be ultimate but just preliminary. Consultation with a cardiac surgeon or child neurologist should be considered, though perhaps merely the preparation of the pregnant woman by an expert physician would be enough.

Changing the haemodynamics of the fetus in the 3^rd^ trimester forms the basis of a new classification used in prenatal cardiology, which differs from that used in pediatric cardiology [3, 9, 11].

Case 9 illustrates an unresolved problem when an attempt to remove a foreign body from the fetal heart was made. It is difficult to judge why this risky and experimental procedure was attempted at all. As prenatal cardiology develops, new problems arrive that have not been described in textbooks so far. New methods of treatment are suggested and some centers use experimental therapies. It seems that to judge such experimental treatment, a common registry should be created. In the case described here, treatment based on the only case that had been described does not seem appropriate and more data should be published [32].

In our unit at the IPMHC, invasive procedure, such as decompression in cases of hydrocephalus, uropathies and fluid collections in the body have been performed for some 15 years. The procedures were successful but detrimental for fetuses and such pregnancies frequently led to intrauterine deaths or miscarriages. Also, newborns frequently died and cardiac malformations were detected by an autopsy [33, 34, 35, 36, 37, 38, 39].

It is currently known that intrauterine invasive procedures require prenatal examination, meaning fetal echocardiography to confirm the normal cardiac structure, and post procedure fetal echocardiography to exclude functional abnormalities. If such abnormalities appear after surgery, the fetus requires pharmacotherapy to prevent cardiac insuffciency and prematurity.

Prenatal echocardiography enables diagnosis and prognosis, sometimes unfavorable for either the fetus or newborn. In the latter situation both the pregnant woman and medical personnel should be prepared for the demise of the fetus or newborn [40, 41, 42].

## Conclusions

It is mandatory to develop new multidisciplinary centers of pre- and perinatal cardiology and to organize training for specialists in these fields of medicine.
